# Examining Endothelial Function and Platelet Reactivity in Patients with Depression before and after SSRI Therapy

**DOI:** 10.3389/fpsyt.2016.00018

**Published:** 2016-02-18

**Authors:** Tye Dawood, David A. Barton, Elisabeth A. Lambert, Nina Eikelis, Gavin W. Lambert

**Affiliations:** ^1^Human Neurotransmitters Laboratory, Baker IDI Heart & Diabetes Institute, Melbourne, VIC, Australia; ^2^Monash Alfred Psychiatry Research Centre, Monash University, Melbourne, VIC, Australia; ^3^Department of Physiology, Monash University, Melbourne, VIC, Australia; ^4^Faculty of Medicine, Nursing and Health Sciences, Monash University, Melbourne, VIC, Australia

**Keywords:** affective disorders, antidepressants, major depressive disorder, cellular adhesion molecules, P-selectin, cardiovascular disease

## Abstract

Although it is recognized that patients with major depressive disorder (MDD) are at increased risk of developing cardiovascular disease (CVD) the mechanisms responsible remain unknown. Endothelial dysfunction is one of the first signs of CVD. Using two techniques, flow-mediated dilatation in response to reactive hyperemia and laser Doppler velocimetry with iontophoresis, we examined endothelial function in the forearm before and after serotonin-specific reuptake inhibitor (SSRI) treatment in 31 patients with MDD. Measurement of intercellular adhesion molecule-1, vascular cell adhesion molecule-1, soluble P-selectin, and noradrenaline in plasma was also performed. Prior to treatment, markers of endothelial and vascular function and platelet reactivity were within the normal range. Following SSRI therapy (95 ± 5 days) symptoms of depression were reduced (paired difference between pre- and post-treatment Hamilton rating −18 ± 1, *P* < 0.001) with 19 patients recovered and 4 remitted. There occurred no significant change in markers of endothelial or vascular function following SSRI therapy. The improvement in Hamilton depression rating in response to therapy could be independently predicted by the baseline arterial plasma noradrenaline concentration (*r*^2^ = 0.36, *P* = 0.003). In this cohort of patients with MDD, SSRI therapy did not influence endothelial function or markers of vascular or platelet reactivity. Patient response to SSRI therapy could be predicted by the initial circulating level of noradrenaline, with noradrenaline levels being lower in responders.

## Introduction

The link between cardiovascular disease (CVD) development and major depressive disorder (MDD) is well described (1–3). Importantly, the detrimental effect of MDD on CVD is already evident in young individuals (4). Although the mechanisms linking MDD and CVD development remain to be unequivocally determined previous studies have provided some evidence of sympathetic nervous activation, endothelial dysfunction, and increased platelet reactivity in patients with MDD. Together, or in concert with other lifestyle factors, these physiological processes may provide an environment that in depressed individuals may predispose them to increased CVD risk.

Endothelial dysfunction is one of the earliest signs of CVD development. Studies investigating endothelial dysfunction in patients with MDD have yielded conflicting results. Symptoms of anxiety and depression in women, which were measured before and after menopause, were associated with endothelial dysfunction 5 years post-menopause (5). In young adults with untreated depression, Rajagopalan and colleagues also reported endothelial dysfunction in patients with depression (6), whereas Garcia et al. noted that reduced concentration of nitric oxide metabolites in plasma were not associated with impaired endothelial function in young, drug-naive patients with first onset depression (7). Although endothelial function was shown to be improved in patients with coronary heart disease and symptoms of depression following 20 weeks of sertraline treatment (8), a shorter period of therapy with either of these, sertraline, fluoxetine, or paroxetine, in women with no history of CVD was associated with no improvement in endothelial function (9). Given the equivocal nature of these results, in this study, we aimed to examine endothelial function, in otherwise healthy patients with MDD, prior to and following serotonin-specific reuptake inhibitor (SSRI) treatment using two different techniques, flow-mediated dilatation in response to reactive hyperemia to examine endothelial function in large vessels in the forearm, and, in small vessels, laser Doppler velocimetry (LDV) with iontophoresis of acetylcholine (ACh) and sodium nitroprusside (SNP) (10). Blood samples were obtained to examine markers of vascular function and platelet reactivity.

## Materials and Methods

This study formed part of a larger study assessing cardiovascular risk prior to and following SSRI treatment in patients with MDD (11, 12). Data obtained from 31 patients (19 females and 12 males) with MDD forms the basis of this report. The Alfred Hospital Ethics Committee approved the research protocol and participants gave written informed consent prior to commencing the study. Participants were recruited *via* local advertisement or following referral from general physicians. Following initial telephone screening, subjects underwent a physical examination. Exclusion criteria included comorbid medical conditions, such as pre-existing heart disease, type 1 diabetes, medicated hypertension, epilepsy, bleeding disorders, alcohol/drug dependence, infectious blood diseases, comorbid psychotic disorders, eating disorders, mental retardation, dementia and personality disorders, and the use of any medication including anti-coagulants. Patients were either newly diagnosed or currently untreated after a relapse and had not been taking antidepressants, including herbal remedies, or benzodiazepines for at least 4 weeks prior to the study (5 weeks if they had been on fluoxetine). No patients presented with psychotic symptoms. Patients having previously failed to respond to treatment with a SSRI at the maximum tolerated dose for at least 4 weeks were not included in the study. Investigations were performed at baseline in unmedicated patients and following at least 12 weeks SSRI therapy (*n* = 22 citalopram, *n* = 4 sertraline, *n* = 3 fluvoxamine, and *n* = 2 fluoxetine).

All participants had a psychiatric interview using the full Mini International Neuropsychiatric Interview (MINI) to ensure that they met DSM-IV diagnostic criteria for MDD. Hamilton depression (HAM-D) scale and Beck depression inventory (BDI-II) were used to monitor progress. Standard cut offs for recovery and remission, that is, 50% drop in HAM-D and BDI-II score for remission, and a score of <8 in HAM-D and BDI-II to signify recovery, were used. Spielberger’s state and trait anxiety inventories were used to assess the level of underlying anxiety. Eligibility for entry was determined by HAM-D > 18, BDI-II > 18, and major depression diagnosis on MINI and deemed to have a significant major depression as the primary illness at psychiatric interview. Only patients diagnosed on the MINI with major depression or major depression with melancholia according to ICD 10 and DSM-IV criteria were included. Patients with comorbid panic or anxiety disorders were included in the study, if the primary diagnosis was depression and any panic/anxiety was secondary to their depression.

Measurement of endothelial function was assessed by strain gage plethysmography of the forearm and LDV with direct current iontophoresis. Strain gage plethysmography measures forearm blood flow in response to reactive hyperemia in resistance vessels (13). In the current study, venous occlusion pressure was set at 50 mmHg, while blood flow to the wrist and hand were excluded by application of a pediatric blood pressure cuff to the wrist raised to suprasystolic blood pressure of 200 mmHg. The venous cuff, positioned around the upper dominant arm, was plugged into an automatic pneumatic inflator (Hokanson E20 rapid cuff inflator, Washington, DC, USA). Changes in forearm volume were measured using an indium–gallium filled double-stranded strain gage (Hokanson, Washington, DC, USA) placed around the widest part of the forearm, connected to a plethysmograph (Hokanson EC6, Washington, DC, USA). Baseline flow measurements were obtained by averaging three to five measurements of 10-s cycles of successive inflation/deflation of the upper arm cuff. Arterial blood flow occlusion was achieved by inflating the upper arm cuff to 150 mmHg, or approximately 20 mmHg above systolic blood pressure, for 5 min (termed reactive hyperemia). Following reactive hyperemia, the wrist cuff was once again inflated to supraphysiological levels, and the upper cuff was suddenly deflated and the cycles of venous compressions immediately resumed until blood flow returned to baseline levels. As a positive control, 300 μg glyceryl trinitrate [GTN (Sigma, VIC, Australia)] was administered sublingually before 5-min reactive hyperemia was initiated. Glyceryl trinitrate is a nitric oxide donor; therefore, it is used as an endothelium-independent marker, providing a comparison with endothelium-dependent forearm blood flow. Data were acquired, recorded, and analyzed using MacLab/2e (ADInstruments, NSW, Australia), and the computer program Chart v4.1.2 (Chart for Windows, ADInstruments). Results were expressed as % change from baseline.

Subcutaneous vascular reactivity was measured using LDV with direct current iontophoresis. A dual channel Moor DT4 laser Doppler flowmeter (Moor Instruments, England) was used to measure erythrocyte flux. This was achieved using a helium–neon infrared light from a laser, with a wavelength of 632.8 nm, *via* a fiber optic probe, which measures changes in the frequency shift that is produced by the scatter of photons from erythrocytes 1–2 mm below the skin surface. The Doppler shift magnitude is directly proportional to erythrocyte flux (14). The subject was seated with his/her right arm extended across a hospital table, in a holding apparatus to eliminate arm movement, with the anterior surface facing the flowmeter. Polyvinyl chloride (PVC) chambers, specially made by us, with a central reservoir of 12 mm in diameter, or 0.5 ml volume, were placed on the forearm with double-stick disks (3M Health Care, ON, Canada). The chambers were positioned in a manner likely to avoid any apparent broken skin or hair growth. The subject was asked to sit in the same position for 10 min prior to, and throughout the procedure, to maintain basal blood flow levels in the vasculature. Assessment of erythrocyte flux was recorded on a computer using the Moor Instruments laser Doppler perfusion measurement package V3.01. Each of the 4 PVC chambers was scanned consecutively 25 times over a period of approximately 7 min incorporating 2 baseline scans, 30 s of perfusion, and the resultant vasodilatory response. Investigations of vascular endothelial function using this or similar procedures have traditionally utilized ACh to assess endothelium-dependent vasodilation and SNP for endothelium-independent vasodilation (15–18). ACh mediates endothelium-dependent vasodilation *via* interactions with nicotinic receptors present on endothelial cells. This results in NO release from the endothelium and subsequent vasodilatation. SNP is a nitric oxide donor that induces vasodilation by acting directly on the smooth muscle cells. Therefore, in the present study, endothelium-dependent vasodilation was measured by the iontophoresis of Ach (BDH Chemicals, UK) andendothelium-independent vasodilation, the positive control, was assessed by the iontophoresis of SNP (Davis Bull Laboratories, Australia). Solutions of ACh and SNP were prepared, at least 24 h prior to the study, in methyl cellulose gel (10% w/v) (Sigma, MO, USA). To complete the circuit, a metal clip was placed onto a platinum wire, which lined the chamber interior on the skin/chamber interface. An iontophoresis controller provided a direct current for perfusing the gel. An anodal current of 0.1 mA and a cathodal current of 0.1 mA were used to iontophorese ACh (10 mg/ml) and SNP (10 mg/ml), respectively, for 30 s. The speed with which a drug can be delivered is influenced by its molecular weight (19) and the degree of ionization of the drug (20). The coefficient of variation for measuring subcutaneous vascular reactivity determined by this method is 0.15 ± 0.05 (10). Responses to gel perfusion were measured as a frequency shift by the laser Doppler flowmeter and analyzed by the Moor Instruments laser Doppler perfusion computer package V3.01. The images displayed different colors, representing the degree of blood flux, on a scale of 250 U. Area under the curve was measured using Microsoft Excel (Microsoft Office 2000, Washington, DC, USA) to quantify each response. Data are represented as arbitrary perfusion units.

Following completion of the assessment of endothelial function an arterial line was placed aseptically in the brachial or radial artery by a cardiologist, as previously described (11). After at least 20 min of supine rest, a blood sample (30 ml) was obtained for the determination of markers of vascular and platelet function, including noradrenaline, soluble P-selectin (P-selectin) soluble intercellular adhesion molecule-1 (ICAM-1), and soluble vascular cell adhesion molecule-1 (VCAM-1). Previous studies have demonstrated that elevated levels of these compounds may be independent risk factors for the development of atherosclerosis and CVD (21–23). The sample was collected in EDTA, lithium heparin, and EGTA-reduced glutathione tubes, stored on ice, centrifuged, and the plasma stored at −80°C until assayed. Noradrenaline was determined by high performance liquid chromatography with Coulometric detection, as previously described (24). Soluble P-selectin, ICAM, and VCAM were determined using commercially available kits (R&D Systems, Minneapolis, MN, USA) according to the manufacturer’s instructions.

### Statistical Analyses

Statistical analysis was performed using IBM SPSS Statistics Version 22. Data are reported as mean ± SEM unless specified otherwise. Associations between variables were examined using Pearson’s product moment correlation coefficient and linear regression analysis. The effect of SSRI treatment was examined using a paired Student’s *t*-test. Non-Gaussian data were logarithmically transformed prior to analysis. A two-tailed value of *P* < 0.05 (with consideration of Bonferroni adjustment for multiple comparisons of biochemical variables) was deemed significant.

## Results

Demographic data, the level of depression and anxiety, and markers of endothelial and vascular function of participants are presented in Table 1. There was no difference in the level of depression or anxiety between genders and no association between age and BMI and the level of depression, as indicated from both the Hamilton and BDI scores. Older participants experienced a lower state anxiety level during the investigation (*r* = −0.49, *P* = 0.005). After controlling for age, there was a trend for an association between systolic blood pressure and state anxiety (*r* = −0.33, *P* = 0.09). There occurred no association between the level of depression or anxiety and markers of vascular function. The levels of noradrenaline and P-selectin in plasma were positively linked (*r* = 0.83, *P* < 0.001).

**Table 1:** **Participant demographics, endothelial function and markers of vascular function prior to and following SSRI therapy**.

	Baseline	Post SSRI	*P*
Age (years)	43.9 ± 2.0		
HAM-D	25 ± 1	8 ± 1	<0.001
BDI	29 ± 1	10 ± 1	<0.001
State	58 ± 2	38 ± 2	<0.001
Trait	64 ± 1	45 ± 2	<0.001
BMI (kg/m^2^)	26.0 ± 0.8	24.9 ± 0.8	0.06
Systolic BP (mmHg)	134 ± 2	133 ± 3	0.75
Diastolic BP (mmHg)	70 ± 2	69 ± 2	0.47
Heart rate (beats/min)	67 ± 2	64 ± 2	0.01
Plasma noradrenaline (pg/ml)	307 ± 42	201 ± 26	0.006
ICAM (ng/ml)	233 ± 15	234 ± 14	0.48
VCAM (ng/ml)	497 ± 26	483 ± 30	0.69
P-selectin (ng/ml)	56 ± 14	44 ± 7	0.40
Forearm blood flow (% change)			
Endothelium dependent (% change)	1158 ± 122	1017 ± 163	0.43
Endothelium independent	1319 ± 417	1196 ± 177	0.9
Microiontophoresis (arbitrary units)			
Acetylcholine	83 ± 16	81 ± 9	0.63
Sodium nitroprusside	91 ± 12	82 ± 11	0.56

Following SSRI therapy (95 ± 5 days), symptoms of depression and anxiety were markedly reduced (paired difference between pre- and post-treatment: Hamilton rating −18 ± 1, BDI −19 ± 2, trait anxiety −19 ± 2, state anxiety −19 ± 3, and all *P* < 0.001). Four participants did not return for the follow-up visit. Using standard cut offs for recovery and remission, that is, a 50% drop in Ham-D for remission, and a score of <8 in HAM-D to signify recovery, 19 were recovered, and 4 were remitted. The change in Ham-D was <50% in four participants.

Treatment with an SSRI did not significantly alter strain gage plethysmography determinations of endothelium-dependent (1017 ± 163% change from baseline, *P* = 0.43) or endothelium-independent vasodilation (1196 ± 177% change from baseline, *P* = 0.9). Similarly, SSRI therapy did not significantly influence vascular response to iontophoretically administered ACh (81 ± 9 arbitrary units) or SNP (82 ± 11 arbitrary units) in the forearm vasculature. Although the plasma noradrenaline concentration was reduced significantly following SSRI treatment (201 ± 26 pg/ml, *P* = 0.006), there occurred no change in the plasma concentration of either of these, ICAM (234 ± 14 ng/ml), VCAM (483 ± 30 ng/ml), or P-selectin (44 ± 7 ng/ml), in response to SSRIs.

Participants who were recovered, with a Ham-D score <8 following SSRI therapy, had lower trait anxiety (62 ± 2 vs. 66 ± 3, *P* = 0.02) and lower plasma noradrenaline concentration (245 ± 31 vs. 512 ± 136 pg/ml, *P* = 0.03) at baseline. There was a trend for plasma P-selectin to be higher in those subjects who did not respond as well to SSRI treatment (39.7 ± 3.3 vs. 95 ± 42 ng/ml, *P* = 0.06). The change in Ham-D score was correlated with the baseline plasma noradrenaline concentration (*r* = −0.59, *P* = 0.003), P-selectin (*r* = −0.47, *P* = 0.02), and VCAM (−0.47, *P* = 0.02) concentrations and the change in plasma noradrenaline level after SSRI therapy (*r* = 0.44, *P* = 0.03). Similar findings were evident when the improvement in depression was estimated using % change in the Ham-D rating, with baseline trait anxiety (*r* = −0.42, *P* = 0.03), baseline plasma noradrenaline (*r* = −0.66, *P* = 0.001), and P-selectin (*r* = −0.53, *P* = 0.006) and change in plasma noradrenaline (*r* = 0.51, *P* = 0.01) being significant. Using forward stepwise multiple regression analysis, the change in Ham-D rating following SSRI treatment could be independently predicted by the baseline plasma noradrenaline concentration (*r*^2^ = 0.36, *P* = 0.003). We found no differential effect between citalopram and other SSRI medications on any of the parameters examined.

## Discussion

We assessed endothelial function using the methods of strain gage plethysmography and LDV with direct current iontophoresis. Contrary to some previous reports, we found that antidepressant therapy was not associated with any change in endothelial function or of markers of vascular function or platelet reactivity. Interestingly, there occurred a significant reduction in the plasma level of noradrenaline following SSRI treatment and that the improvement in Ham-D score following SSRI therapy was associated with the plasma noradrenaline level at baseline. Although the clinical significance of these findings remains uncertain, our observations do not support a role of endothelial dysfunction as an etiological factor in the increased cardiac risk associated with MDD.

There exist conflicting reports on endothelial function in patients with MDD (6, 7). Rajagopalan and colleagues documented endothelial dysfunction in young patients with depression (6), whereas Garcia et al. reported that endothelial function did not differ between young healthy subjects and patients with first onset depression, despite there occurring a reduced plasma level of metabolites of nitric oxide (7). In the present report, we found no change in measures of endothelial function in both the large vessels and resistance vessels following SSRI therapy. This observation contrasts with some reports that documented a worsening of endothelial function in patients with MDD following SSRI therapy (25). Broadley et al. demonstrated that depressed patients undergoing effective therapy, in fact, displayed an impairment in endothelial function (25). Conversely, Hantsoo et al. demonstrated that SSRI treatment exerted little effect on endothelial function or measures of platelet reactivity in women with depression (9). Whether the discordance between these results and ours is related to differences in medications used, severity of illness, or age and gender of participants remains unknown. Aging is associated with a progressive decline in endothelial function (26, 27) and gender, due at least in part to the difference in vessel size (28), and phase of the menstrual cycle (29, 30) has been shown to influence endothelial function.

We found no effect of SSRI therapy on the plasma level of ICAM, VCAM, or soluble P-selectin. Previous studies have indicated that patients with MDD may be susceptible to platelet activation, and possibly predisposing them to increased CVD risk (31). Whether the level of soluble P-selectin in plasma provides a reliable indicator of actual platelet activation may be open to question (32). Nevertheless, our observation of an association between plasma noradrenaline and P-selectin is intriguing. Elevated levels of noradrenaline in plasma enhance platelet aggregability and platelet secretion *in vivo* in healthy humans (33). Whether heightened sympathetic nervous activity, which may be present in around one-third of patients with MDD (11), is associated with platelet reactivity deserves further attention. Of note also is our observation that baseline arterial plasma noradrenaline concentration predicted the improvement in depression following SSRI therapy. Although the majority of noradrenaline in plasma is derived from sympathetic nerves, it is important to note that the sympathetic nervous system is of course regulated centrally. Determining the central determinants of high sympathetic tone in patients with MDD may give insight into the neuropathology underlying the condition.

There are a number of limitations to our study which should be noted. We did not include a placebo control group as we thought it unethical to withhold therapy from significantly ill individuals. Hence, it remains unknown whether our patients, in fact, displayed signs of endothelial dysfunction prior to therapy. Although we did not measure levels of ICAM, VCAM, or P-selectin in healthy subjects, the concentrations determined seem to be in the range reported for control subjects by others (21, 34, 35). It is perhaps not surprising therefore that SSRI therapy was without an effect on these parameters. Endothelial function was assessed using two methods, flow-mediated dilatation in response to reactive hyperemia and LDV with direct current iontophoresis. Although these methods are widely used, particularly in cardiovascular research, gold standard methods typically involve intracoronary or intrabrachial arterial infusion of vasoactive substances (36).

In conclusion, examination of endothelial function, determined by plethysmography of the forearm and LDV, did not reveal differences between endothelium-dependent blood flow and endothelium-independent blood flow in patients with MDD prior to or following SSRI treatment. We found no effect of SSRI therapy on the plasma level of ICAM, VCAM, or P-selectin. The baseline arterial plasma noradrenaline level was predictive of the improvement in severity of depression following SSRI treatment. Whether endothelial dysfunction is impaired and is associated with increased cardiac risk in patients with MDD remains unknown.

## Author Contributions

TD: conceived and designed research; acquired, analyzed, and interpreted data; drafted manuscript. DB: conceived and designed research; acquired data; approved manuscript. EL: conceived and designed research; acquired data; approved manuscript. NE: acquired, analyzed, and interpreted data; approved manuscript. GL: conceived and designed research; acquired, analyzed, and interpreted data; drafted manuscript.

## Conflict of Interest Statement

Lundbeck Australia provided cipramil for use in this study.
